# Combination of medical and surgical management in successful treatment of caesarean scar pregnancy: a case report series

**DOI:** 10.1186/s12884-020-03237-8

**Published:** 2020-10-13

**Authors:** Anda Pristavu, Angela Vinturache, Elena Mihalceanu, Radu Pintilie, Mircea Onofriescu, Demetra Socolov

**Affiliations:** 1 Cuza-Voda Obstetrics and Gynecology University Hospital, Grigore T.Popa University of Medicine and Pharmacy, Iasi, Romania; 2 Department of Obstetrics & Gynaecology, Women’s Centre, John Radcliffe University Hospital, Oxford University Hospitals NHS Foundation Trust, Headley Way, Oxford, OX3 9DU UK

**Keywords:** Caesarean scar pregnancy, Transvaginal ultrasound, Hormone chorionic gonadotropin, Methotrexate, Mifepristone, Curettage, Case report

## Abstract

**Background:**

There is no clear consensus on the management of caesarean scar pregnancy (CSP), a complex and life-threatening condition. The objective of this study was to present a novel approach to management of CSP that combines medical therapy of multidose methotrexate and mifepristone with active surgical management by uterine curettage and consecutive local haemostasis.

**Case presentation:**

We report on a prospective case series of six women with first trimester pregnancy, in whom the diagnosis of CSP was confirmed by 2D and color Doppler transvaginal ultrasound and serial hormone chorionic gonadotropin (hCG) testing. Women were between 23 and 36 years old and had at least one previous delivery by caesarean. At admission, gestational age ranged between 6 to 14 weeks, and serum hCG levels between 397 and 23,000 mUI/ml. Upon decision of pregnancy termination, medical management was undertaken in all cases and 1 mg/kg systemic Methotrexate was administered between 1 and 5 daily doses. Mifepristone was part of the treatment in cases with live pregnancy. Surgical management was employed for the cases were an embryo was seen by ultrasound, being prompted by inadequate response to Methotrexate and/or signs of miscarriage with vaginal bleeding. Curettage combined with local isthmic balloon or vaginal pack tamponade prevented further complications. High treatment rates with preservation of fertility was achieved in all patients except one who underwent hysterectomy for invasive placentation. Ultrasound and hCG levels surveillance ensured that the resolution of pregnancy was achieved.

**Conclusion:**

Women with history of delivery by caesarean section should be carefully monitored in future pregnancies for prompt diagnosis of CSP. Early diagnosis of CSP allows selection of successful conservative therapy. Through this case series we contribute with our experience to the body of knowledge about the management of this serious complication of early pregnancy.

## Background

The caesarean scar pregnancy (CSP) is a rare complication in women who had a previous caesarean section (CS). In CSP, gestational sac is implanted in the hysterotomy scar. Although classified as a type of ectopic pregnancy, CSP is not an ectopic pregnancy by definition as the most part of the gestation, including the placenta, are localised in the CS scar but face and develops towards the uterine cavity to become part of it [1]. .CSP is a life-threatening condition due to the increased risk of rupture and excessive bleeding that may endanger woman’s life and/or compromise future fertility. Diagnosed early, treatment options can preserve the uterus and subsequent fertility. Recent research has suggested that CSP is a precursor of morbidly adherent placenta [2].

CS is on the rise worldwide. With the escalating CS rates, there are raising concerns over the upright trends in the short- and long-term risks and costs [3], including the increase in rare complications such as uterine rupture, placental disorders, isthmocele, and CSP. The risk of such complications increases with the number of prior CSs, although other factors such as previous dilatation and curettage (D&C) might be an associated risk factor [4–6].

The incidence of CSP was reported in the range of 0.04 to 0.05% (1/1800 to 1/2216) of all pregnancies [1, 4, 5] and 0.15% in women who had previous CSs [7]. More than half (~ 52%) of the CSP cases occur in women which had only one prior CS [8]. Because of its rarity, there is no consensus on best management. Both the diagnosis and the treatment of CSP remain challenging. Low index of suspicion of CSP leads to delayed diagnosis or findings misinterpretation. Ultrasound scan, Doppler examination, and magnetic resonance imaging (MRI) are all useful in early detection of CSP. Several sonographic criteria have been developed to aid with the timely diagnosis [9]. . A retrospective analysis of 2037 CSP cases identified as many as 14 therapy models [10], including but not limited to expectant management, systemic or local administration of Methotrexate, D&C, local resection of the ectopic gestational mass by minimally invasive surgery (hysteroscopy, laparoscopy), or total hysterectomy. Systematic reviews of management options of CSP support an interventional rather than a medical approach, although no conclusion was reached regarding a specific method [11, 12].

In this article, we present a case series of CSP, prospectively collected at our institution, reporting on the clinical, biological, and sonographic elements that led to early diagnosis and management. We discuss the approach to management of these patients based on the clinical and technical means available to us, through the perspective of published literature on the subject. Our study has the potential to contribute further evidence towards an international standardized protocol for management of CSP.

## Case presentation

This case report series include six patients who presented to our hospital, a tertiary referral centre, over a period of 3 years, from 2017 to 2019, with history of several weeks of amenorrhea, vaginal bleeding, and abdominal pain. Women included in this study had a history of one or more previous CSs, a positive hCG suggestive of pregnancy, and underwent clinical assessment aligned to our clinical protocols for early pregnancy complications. All women were assessed by gynaecologists trained in transvaginal ultrasound in early pregnancy. A diagnosis of CSP was suspected in all cases based on the clinical, biological, and sonographic features.

A complete history was taken at the initial presentation, and the demographic and clinical details as well as the laboratory values and sonographic findings were entered in the electronic medical records database and were extracted for this study in an Excel datasheet. This file also included the clinical examination that was carried out by the attending physician and included both, a speculum and bimanual vaginal examination. A blood sample was collected from each woman at admission to ascertain the complete blood count, blood group type, and hCG levels. The serum hCG levels were measured using Immulite 1000 Immunoassay system (Siemens Healthcare GmbH, Germany). The laboratory hCG reference values are presented in the legend of Fig. 3. The monitoring of hCG levels was performed at 48 h interval until hospital discharge, then weekly, with adjustment in the frequency of measurements to specific conditions (i.e. the health status of the patient changed). After hospital discharge, the patients attended periodic monitoring of hCG levels and ultrasound surveillance either in our centre or at the referral centre.

The diagnosis of pregnancy on SC scar was done by two-dimensional transvaginal ultrasound and color Doppler. The transvaginal ultrasound (TVUS) examination was indicated at the initial presentation to assess the viability and location of the pregnancy. All women included in this study met the TVUS criteria for a CSP diagnosis as previously published [1, 9, 13–15].

### Diagnostic criteria of CSP

**Fig. 1:**
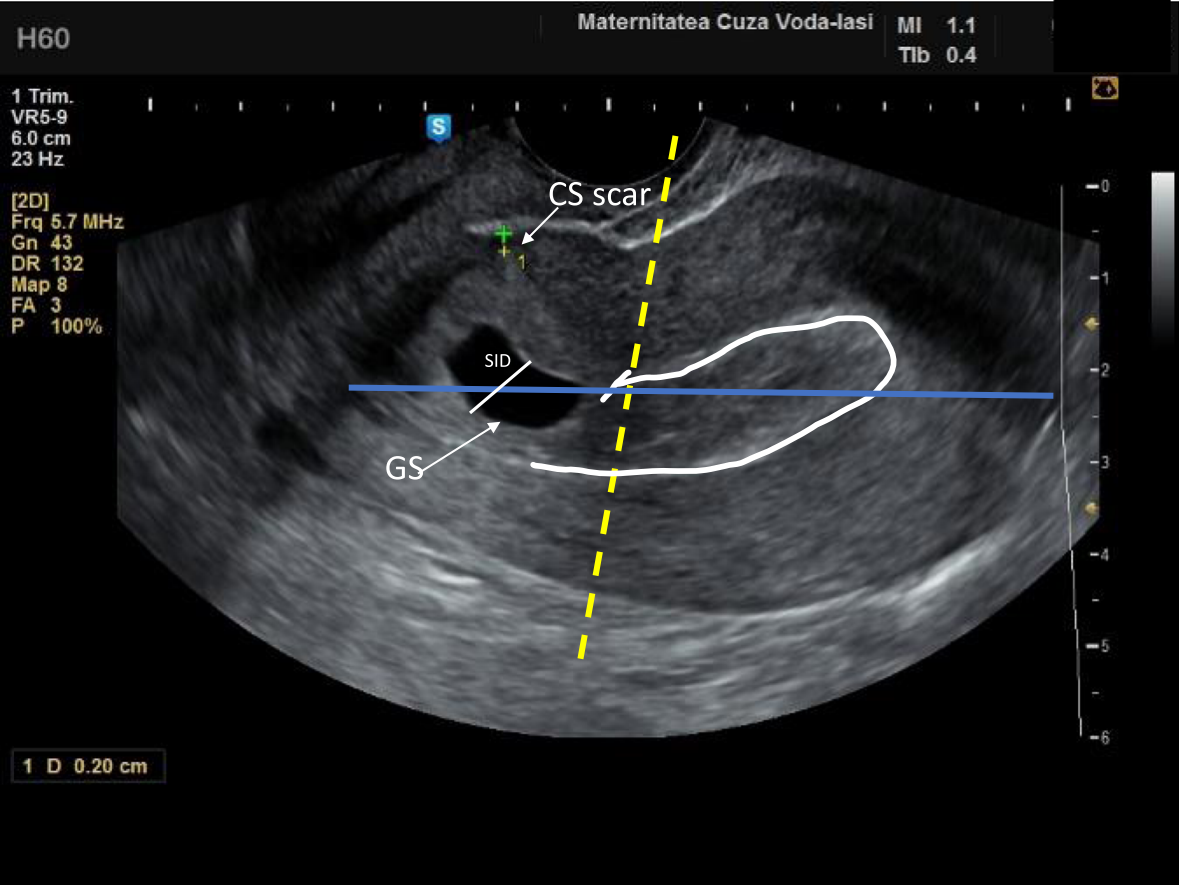
Sonographic differential diagnosis between intrauterine pregnancy and caesarean section scar pregnancy in the early first trimester. Interrupted yellow line on a sagittal section of the uterus – imaginary line dividing uterus in two portions described by Timor-Tritsch et al. [9]. Continuous blue line on a sagittal section of the uterus – imaginary endometrial line connecting internal cervical os with the uterine fundus described by Cali et al. [14]. The gestational sac is situated below the yellow line, implanted at the level of CS scar. Less than two thirds of the SID line are above the endometrial line, towards the anterior uterine wall. The uterine cavity is empty. The myometrial layer thickness is 2 mm. CS, caesarean section; GS, gestational sac; SID, superior-inferior diameter of gestational sac

**Fig. 2:**
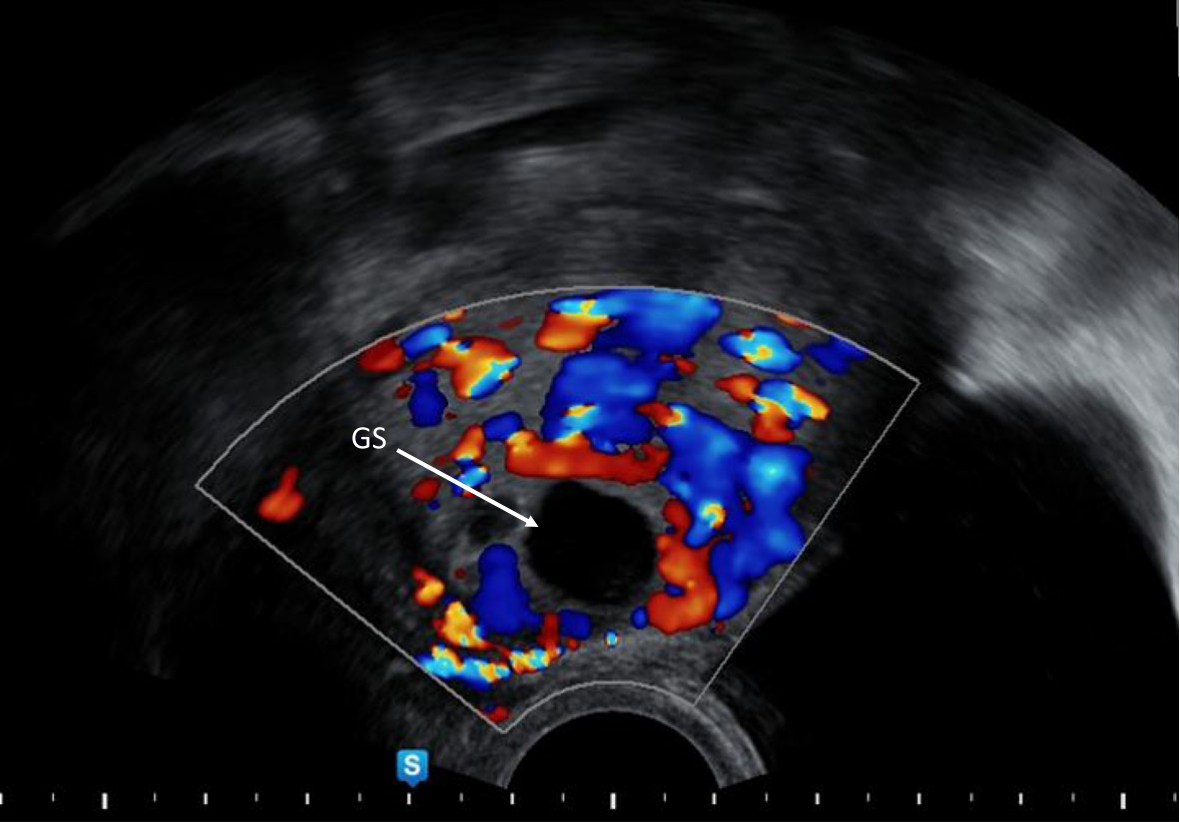
Sonographic diagnosis of caesarean section scar pregnancy in the early first trimester. Peritrophoblastic vascularization on ultrasound Doppler. GS, gestational sac

The diagnosis of CSP was sustained by the following signs (illustrated in Fig. 1) that suggested a gestational sac located low in the uterine cavity, with trophoblast inserted into the hysterotomy scar [15]:

- Empty uterine cavity
- Empty and closed endocervical canal
- The presence of an embryonic/foetal pole and/or yolk sac with or without heart activity
- The gestational sac located at the level of internal os filling the visible or presumed site of the previous CS scar [9, 14].
- A thin (1–3 mm) or absent myometrial layer between the gestational sac and the bladder on a sagittal section of the uterus
- Peritrophoblastic hypervascularisation on color Doppler examination **(**Fig. 2**)**
- Location of the placenta/vascular supply of the gestational sac in the niche of the previous SC scar [13].

The TVUS principles established by Timor-Trish et al. and the crossover sign identified by Cali et al. were used to confirm the location GS and the diagnosis of CSP in all six cases [14] (Fig. 1). A Voluson E8 ultrasound system (GE Healthcare) with a transvaginal IC5–9-D probe (frequency 4.0–9.0 MHz) and a transabdominal RAB6D probe (2.0–8.0 MHz) were used for CSP diagnostic. MRI to confirm CSP was not required for any of the presentations. MRI was used to confirm in one case a morbidly invasive placenta, where a risk of uterine rupture was suspected.

### Management protocol

All patients were hospitalized for treatment. Multidose systemic Methotrexate at 1 mg/kg/dose was first line of management proposed. In patients with live pregnancy, Mifepristone was added to shorten the time until embryo’s death and potentially reduce the dose of Methotrexate. For cases were clinical signs of miscarriage were present or the immediate response to Methotrexate was inadequate with persistence of trophoblastic vascularization, surgical management was initiated, which consisted in curettage with preventative local haemostasis with Foley catheter or surgical uterine sponge or vaginal pack, as clinically indicated.

For women who received Methotrexate treatment, liver and renal function tests completed the laboratory panel, whereas for women who underwent surgical management, blood and Rh type and crossmatch was done (data on these tests not provided). Before initiation of Methotrexate treatment, a thorough history was taken from every patient to identify presence of any contraindications to treatment. These patients underwent periodic testing of renal and liver function. There were no contraindications or complications of Methotrexate treatment in any of our patients. Additional therapy with folinic acid was given to prevent the toxic effects of Methotrexate. The full blood count was rechecked the day after medical and surgical management.

This study was approved by the hospital’s ethics committee. Signed informed consent was obtained from all patients before treatment. The form signed by each patient included consent for CSP treatment modalities, medical and/or surgical, including risk and complications of each, as well as the risk of pregnancy preservation, possible hysterectomy, transfusion of blood products, loss of fertility and death. The patients also sign consent for research and publication, including permission to publish anonymised clinical information and images.

### Summary of clinical findings and management

Table 1 presents the clinical and biological characteristics of the patients. The women were between 23 and 36 years old and had an average of 2 (range 1–3) previous CSs. The average parity was 2.2 (range 1–5), with a total number of pregnancies between 2 and 7 (average 3.8). Mean gestational age at the time of diagnosis was 7.6 weeks (range 5–14 weeks).

**Table 1:** Clinical and biological characteristics of the patients included in the study

Patient	Age (years)	Gestation	Parity	Miscarriages/termination of pregnancy	Previous CSs	Previous vaginal deliveries	Amenorrhea	Gestational age at diagnosis	ß-hCG levels on admission (mIU/mL)
**1**	**36**	**3**	**2**	**0**	**2**	**0**	**6**	**6**	**23,100**
**2**	**32**	**6**	**4**	**1**	**4**	**0**	**8**	**6**	**13,229**
**3**	**31**	**2**	**1**	**0**	**1**	**0**	**5**	**5**	**19,148**
**4**	**32**	**4**	**3**	**0**	**3**	**0**	**12**	**12**	**1549**
**5**	**23**	**3**	**2**	**0**	**2**	**0**	**7**	**9**	**4000**
**6**	**28**	**2**	**1**	**0**	**1**	**0**	**14**	**8**	**397**

*Abbreviations*: *CS* caesarean section, *hCG* hormone chorionic gonadotroph

The clinical findings are described in Table 2. All women presented at admission the three cardinal symptoms: vaginal bleeding, lower abdominal pain, and amenorrhoea. All women had a physical exam at admission that included vital signs and vaginal examination, speculum and bimanual exam.

**Table 2:** Clinical and US findings at presentation for the 6 patients of the case series diagnosed with CSP

Patient	TVUS findings: Uterus	TVUS findings: Gestational sac	TVUS findings: Myometrium at implantation site	Physical examination
**1**	Uterus size 59/58/65 mm Endometrium thickness 17 mm Empty uterine cavity	Gestational sac 32 mm No embryo seen	Gap in the myometrium of the anterior wall at the level of the CS scar, thickness of the myometrial wall 2.0 mm, no free fluid	Changes of pregnancy to vaginal mucosa, small quantity of dark blood in the vagina, closed cervix Soft cervix, closed os, slightly enlarged uterus, tender to palpation
**2**	Uterus size 57/55/62 mm Endometrium thickness 28 mm Empty uterine cavity	Gestational sac 20 mm Yolk sac present Embryo present Embryo heart rate not visualised	Gap in the myometrium of the anterior wall at the level of the CS scar, thickness of the myometrial wall 2.6 mm, no free fluid	Vulvovaginal mucosa with changes of pregnancy, moderate amount of blood in the vagina, cervix with os closed, uterus of 6 weeks size, non-tender to palpation
**3**	Uterus size 69/48/65 mm Endometrium thickness 12 mm Empty uterine cavity	Gestational sac 20.3 mm Embryo present Embryo cardiac activity present (96 b/min)	Gap in the myometrium of the anterior wall at the level of the CS scar, thickness of the myometrial wall 2.0 mm, no free fluid	Changes of pregnancy to vaginal mucosa, moderate amount of dark blood in the vagina, closed cervix Soft cervix, closed os, uterus slightly enlarged of approximately 5 weeks, tender to palpation, mild cervical excitation excitation
**4**	Uterus size of 59/58/65 mm Empty uterine cavity	Gestational sac present. Yolk sac was seen day 3 from admission, before starting medical manag. No embryo seen	Gap in the myometrium of the anterior wall at the level of the CS scar, thickness of the myometrial wall 2.6 mm, no free fluid	Vulvovaginal mucosa with changes of pregnancy, small amount of blood in the vagina, closed cervical os, slightly enlarged uterus, non-tender to palpation
**5**	Uterus size 58/56/69 mm Empty uterine cavity	Gestational sac 17/11 mm An embryo with CRL 4 mm was seen, no cardiac activity was present	Gap in the myometrium of the anterior wall at the level of the CS scar, thickness of the myometrial wall less than 3 mm, no free fluid	Changes of pregnancy, fresh blood in the vagina in moderate amount, cervix open, cervical excitation present, uterus increased in size approx. 8 weeks, tender
**6**	Uterus size 110/68/70 mm Empty uterine cavity	Irregular gestational sac of 23.3 mm with an embryo with CRL 14.8 mm. No cardiac activity was present	Gestational sac protruding into the anterior uterine wall at the isthmus.	No fresh blood in the vagina, normal appearance of the cervix with os closed, cervical excitation present, uterus increased in size approximately 8 weeks, very tender when mobilised, adnexa non-palpable bilaterally

*US* ultrasound, *CSP* caesarean scar pregnancy, *TVUS* transvaginal ultrasound

**Fig. 3:**
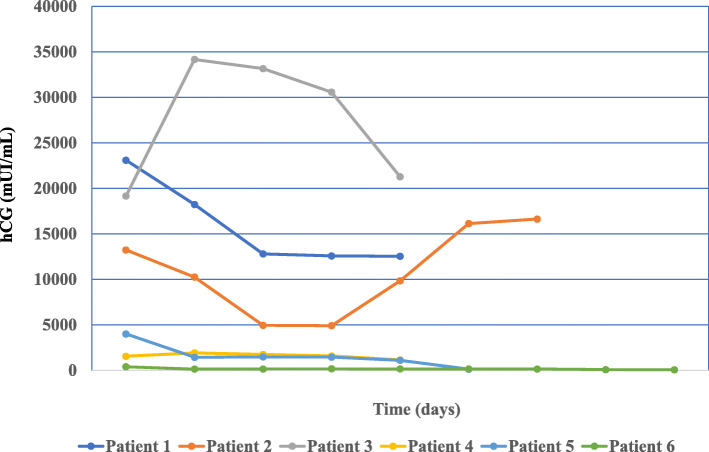
Serial hCG surveillance in 6 patients diagnosed with CSP. Day 1 represents the day of presentation and hospital admission. hCG serum levels were measured using the Immulite 1000 Immunoassay system (Siemens). The laboratory reference values for hCG (mUI/mL) were: non-pregnant, 1–10; week 1–2, 16–156; week 2–3, 101–4870; week 3–4, 1110–31,500; week 4–5, 2560–82,300; week 5–6, 23,100–151,000; week 6–7, 27,300–233,000; week 7–11, 20,900–291,000; week 11–16, 6140–103,000. CSP, caesarean scar pregnancy; hCG, hormone chorionic gonadotroph

As shown in Table 2 all women had a TVUS, the details of which are described in the table. All women had their first US scan the same day of admission. The diameter of the gestational sac measured between 20.0 and 32.0 mm with an average of 27.0 mm. We also reported the yolk sac if seen, measured the cranio-caudal length of the embryo when present, and noted the cardiac activity. The depth of myometrium infiltration and involvement of the scar tissue was determined in all cases and ranged between 1.4 and 2.6 mm. Serum hCG levels at presentation varied widely, ranging between 349 and 23,100 mUI/mL. Figure 3 shows the hCG profile in the serum collected during hospitalization and post discharge surveillance. TVUS follow up at one, six months, or 2 years after treatment showed a normal uterus, with normal appearance of the caesarean scar in all patients.

Table 3 summarises the management, surveillance, and outcomes of each CSP case. The method of treatment chosen for each case was according to the decision made by the individual physicians after patient counselling on the effectiveness of therapy chosen and the risk of hysterectomy. All patients underwent conservative management, including Methotrexate and/or D&C, in order to preserve fertility. Hysterectomy was done for one patient, where was a high index of suspicion of invasive placentation. Five of the patients were administered Methotrexate systemically, 1 g/kg body weight per dose, dispensed up to 5 doses in alternative days. Mifepristone (600 mg oral) was administered prior to Methotrexate to two women, who were diagnosed with live CSP. Suboptimal response to medical treatment prompted further surgical management in four patients. The bleeding ensued at the D&C was promptly managed with insertion of Foley balloon and/or vaginal pack under antibiotic protection. Transfusion of blood products was needed for one patient, who underwent later hysterectomy for suspected accreta.

**Table 3:** Management, complications, and surveillance of the 6 patients diagnosed with CSP included in the case-series report

Patient	Initial treatment	Complications	Management of complications	Hospitalisation (days)	Imagistic surveillance: TVUS and color Doppler	hCG surveillance
**1**	Medical: Methotrexate 1 mg/kg, 5 doses, alternate days	No complications, spontaneous resolution of pregnancy	N/A	9	3-months posttreatment: normal uterus and isthmic caesarean scar	98 days follow up until negative
**2**	Medical: Methotrexate 1 mg/kg, 5 doses, alternate days	Increase hCG levels Persistence of pregnancy and local trophoblastic vascularization	Surgical: D&C and local haemostasis with Foley catheter for 48 h	11	still on surveillance	Decreasing trend of hCG
**3**	Medical: Mifepristone, Methotrexate, 1 mg/kg	Increase hCG levels Spontaneous miscarriage with heavy vaginal bleeding	Surgical, D&C under US guidance haemostasis with uterine-vaginal pack	13	6-months post treatment: normal uterus and uterine scar	< 10
**4**	Medical: Mifepristone, Methotrexate, 1 mg/kg	No complications, spontaneous resolution of pregnancy	N/A	10	1-month post treatment Normal uterus and uterine scar	< 5
**5**	Surgical: D&C	Persistence of vascularised trophoblast	Medical: Methotrexate, 1 mg/kg x 1dose Surgical: D&C and haemostasis with Foley catheter	11	2-years posttreatment Normal appearance of anterior wall of the uterus normal and caesarean scar	Follow up another centre
**6**	Surgical: emergency D&C	Spontaneous miscarriage with heavy vaginal bleeding Persistence of pregnancy, presence of isthmic mass with rich vascularization and possible accreta	Surgical: D&C haemostasis Foley catheter & blood transfusion Surgical: Total abdominal hysterectomy	9	N/A	< 64 mUI/mL

*Abbreviations*: *CSP* caesarean section scar, *hCG* hormone chorionic gonadotroph, *N/A* not applicable. Hospitalization days counts all days as inpatient until treatment completed and discharged to surveillance

Clinical presentations vignettes (Additional File 1) with corresponding images (Additional File 2) are presented as supplementary material. We have included among the figures plenty of US images from admission, during the treatment, and post-treatment surveillance, for instructional purpose.

## Discussion and conclusions

In this study, we assessed the response to systemic Methotrexate, alone or combined with oral Mifepristone, before D&C, and Foley catheter tamponade in the treatment of CSP. The clinical outcomes of five of the six cases of CSP presented in this case-series collected over a period of three years, were similar, assuming the resolution of pregnancy as successful endpoint of the management. We show that, D&C is a reliable management option in management of CSP if used after inhibition of trophoblast growth and the consequent bleeding after the removal of products of conception is promptly prevented with pressure tamponade. We also found that using Mifepristone in live pregnancies is useful to hasten embryo’s demise. Only one of our patients, who was diagnosed in early second trimester with early placenta accreta, required hysterectomy. Emerging evidence point to first trimester CSP as an entity in the continuum leading to morbidly adherent placenta in the third trimester [2, 16–20]. This finding guided the counselling and management of patients diagnosed with CSP in this case series.

The incidence of CSP is increasing worldwide, following the climbing rate of caesarean deliveries [1, 3, 21, 22]. In Romania, although national statistics data on CS are limited, a recent study suggests that the CS rates could be as high as 60% in urban population, in public maternity hospitals [23], and may be even higher in private facilities. A trend in increasing rates of CS was also observed in our unit over the past 10 years (unpublished data). Reported to the number of deliveries in our unit from 2017 to 2019, the incidence of CSP was approximately 1/3000, similar to the incidence from other recent reports [24, 25]. Reported to the number of CS deliveries, the frequency of CSP is about 0.05%. There are two reasons for the reported incidence of SCP in our hospital. We are a tertiary university hospital with referrals of rare complications of early pregnancy from eight counties.. In addition, we are an obstetrics & gynaecology training centre, all the gynaecologists in the unit are trained to perform TVUS and Doppler scans and provide care for early pregnancy.

US, transvaginal and transabdominal US, and color Doppler [26] is first line tool in identifying the markers characteristics of CSP, with a sensitivity of 86.4% [7]. TVUS allowed us positive diagnosis of CSP with high accuracy and characterised the presence, location, and size of gestational sac, presence of the embryo and its cardiac activity, and the relationship between the gestational sac, CS scar, and bladder wall [1, 6, 9]. We used color Doppler functions (i.e impedance, velocity etc) to aid the diagnosis of scar pregnancy, although no clear criteria are yet defined [6, 27]. Only for one women MRI was required to better characterise the trophoblast invasiveness, because of a high clinical suspicion of accreta and/or trophoblastic neoplasia. Our presentation support the use of MRI sparingly, only when other complications are suspected, and should not be routinely employed [1]. The US also proved instrumental for the post therapeutic follow up in all our patients.

Over the past several years an increasing number of publications, reports, and conference presentations on CSP increased the awareness and knowledge on the risks and complications of CSP and its treatment methods. No consensus has been reached and no standard treatment exist to date for CSP [28]. A recent systematic review identified five basic pathways in treatment of CSP: expectant management, medical therapy, surgical intervention, uterine artery embolization, or a combination approach [29–31]. Each method has various levels of success and depends on clinical presentation and resources, patient compliance, and surgeon skills and expertise. The management approach devised by us was based on clinical symptoms, gestational age, pregnancy viability, technical means available, while considering patient’s preferences and aiming to preserve fertility.

Among treatment options, medical management with systemically administered methotrexate with or without local administration of the same or another agent are the most common treatment modalities [31, 32]. Methotrexate is largely used in treatment of tubal and cervical ectopic pregnancy, and its use was extended to CSP, using the same criteria to assess the effectiveness of the treatment [32]. To date, however, there is no protocol for the use of methotrexate in CSP. There is no consensus on the dosage, number of doses needed, interval between doses or knowledge about risk factors or predictors of favourable response. There was no correlation between the hCG initial levels and the favourable response to methotrexate in our study, suggesting that systemic methotrexate could be used in CSP with higher levels of hCG. A recent review supports these findings, showing efficacy of the systemic methotrexate treatment in early CSP, with hCG < 12,000 mIU/ml and absent cardiac activity [33]. Because of short half-life of methotrexate, repeated doses were administered to our patients, on alternative days, at a dose of 1 mg/kg, up to five doses. In a study by Kutuk et al. on the effectiveness of systemic multidose methotrexate treatment in CSP, in 13 patients with CSP and initial hCG levels between 2565 and 36,111 mIU/ml, authors report that between 5.3 and 6 dose cycles of methotrexate were needed alternative days to normalise the hCG levels in CSP with and without cardiac activity, respectively [34]. Kalampokas et al. describe a case of CSP where only 3 dose cycles of 75 mg of methotrexate were needed for a CSP case with viable embryo and hCG of 12,072 mIU/ml to achieve resolution of pregnancy [35]. Similar with our protocol, these authors also used mifepristone 600 mg along with methotrexate [35]. There is mass experience with the use of mifepristone in termination of pregnancy, particularly in viable pregnancies [36, 37]. The use of mifepristone along with methotrexate in medical management of unruptured ectopic pregnancy is less known [38]. It has been suggested that mifepristone may reduce the total doses of methotrexate and accelerates the time to embryo death [35, 39]. For this rationale, we also included 600 mg of oral mifepristone as part of the CSP management protocol.

Surgical management with conservation of fertility was done in all our cases but one. The main objective of this management was to remove products of conception while aiming to prevent and contain massive haemorrhage. Retention of products of conception after CSP treatment is a major concern, as it may adversely influence menstruation and future fertility [40]. In addition, the therapeutic approach of D&C with/without immediate haemostatic measures (i.e Foley catheter balloon, uterine/vaginal pack) intended to conserve the uterus and fertility and maintain woman’s health and quality of life [35]. The D&C was practiced shortly after administration of methotrexate, without awaiting the full response to the drug, this management being supported by previous observations. There are individual reports of unsuccessful treatment of scar pregnancy with systemic methotrexate. In a review of 751 CSP cases, 331 (44.1%) had complications requiring further surgical management. Among 32 different treatment modalities, methotrexate was associated with higher complication rate (62.1%). In this study and others, methotrexate was considered to hinder additional embryonic growth. However, as also observed in our study, the increase in hCG after methotrexate administration is more likely due to hormone release after trophoblastic cytolysis induced by methotrexate [40].

Every method of treatment of CSP carries high risk of excessive haemorrhage. Curettage after medical treatment has a high rate of success and no significant effects on the intraoperative bleeding. The predictors of the risk of bleeding during the procedure are gestational age and the size of gestational sac [41]. The combination of methotrexate and curettage proposed by us is supported by others. For instance, Wang et al. analyses the methotrexate with and without curettage and shows that both therapies could treat the majority of CSP patients successfully, but the combined therapy resulted in a shorter time of therapy and had a more favourable effect [42]. Another study in 45 patients shows that methotrexate administration followed by suction curettage with Foley tamponade was an effective treatment for CSP [43].

There are several limitations to our study. One limitation is the sample size. This limitation is characteristic to the nature of a case series report. This is explained, however, through the rarity and diagnosis challenges of such presentation. Most of our evidence on CSP diagnosis and management comes from similar case reports and case series reports. The vast majority of the studies were retrospective, the management reported being a reflection of the experience and surgical skills of an individual or a group rather than an evidence-based approach. In contrast, the cases from our study were collected prospectively and a formal management plan was followed, although the plan was individually tailored. The management proposed by us was devised for symptomatic patients, reporting pain and persistent vaginal bleeding, and may not be applied to asymptomatic patients. It is also possible that the incidence of CSP may be higher than reported here. It is likely that among the early pregnancies that end up in miscarriages or pregnancy terminations done in private offices there is a certain number of undiagnosed CSP. That is because most CSP do not cross beyond the first trimester and end up in 1st trimester miscarriage. This and the risk of accreta has led to the common practice of offering termination of pregnancy in CSP, similar to other complications of early pregnancy [44, 45].

Serial hCG and TVUS color Doppler are useful in monitoring the treatment and its success, as it appears to be a good correlation between the hCG values and persistence of the trophoblastic flow at the site of an ectopic pregnancy. Doppler examination showing evidence of persistent functional trophoblast was instrumental for this study in surveillance of the response to treatment and decisions to proceed with surgical management. Previous studies have shown a correlation between the Doppler characteristics such as high-velocity, low-impedance, turbulent flow during the initial follow up and the risk of profuse bleeding [1]. However, these parameters were not systematically measured in our study, thus, it is unclear if this information would have been a warning for the physician to not perform a D&C.

The use of curettage in the management of CSP is rather controversial, and the argument is that the procedure may lead to heavy if not catastrophic bleeding [1, 45]. We were able, however, to contain the bleeding and obtain an efficient haemostasis in all cases by prompt intervention. Also, in all cases but one the drop in Hb levels was enough to grant replacement with blood products. Nonetheless, in this case, the copious spontaneous bleeding came from an accreta led to salvage D&C. None of our D&Cs required salvage hysterectomy to contain the bleeding.

There is little scientific data on the risk of recurrence of the condition in future pregnancy, the role of the interval between the previous caesarean delivery and re-occurrence of caesarean scar pregnancy [7, 46]. Few published studies have reported on the low risks or recurrence of SCP, suggesting that implantation into the CS scar is more likely a chance event, than the result of an affinity of a pregnancy for implanting into the scar [47]. We did not follow up our patients long enough to assess their further fertility. Nonetheless, our patients have been counselled on overall good fertility outcomes following a CSP, while being aware of the low risk of CSP recurrence and need for early surveillance in future pregnancies [48].

Despite the experience accumulated by us and others in management of CSP, the evidence to date is not strong enough to be translated in a reliable risk scoring system to guide management. Although a standardised clinical protocol for management is yet be developed, this case series contributes our experience to building the evidence for the management of this complex clinical presentation.

Further studies are warranted to deepen our understanding of the CSP pathophysiology and facilitate development of clinical pathways for this presentation. We endorse the project of an international CSP registry [49] that has been recently proposed with the purpose to collate all the knowledge and produce the best evidence for CSP diagnosis and management.

## Supplementary information

**Additional file 1.** Clinical Vignettes. This file includes detailed description of the six case reports.

**Additional file 2 Fig. S1.** Patient 1. Panel A. Gestational sac present at the level of uterine isthmus, localised within CS scar. Embryo not seen. Panel B. Increased peri-throphoblastic vascularization on color Doppler. Panel C. Gestational sac still present day 5 post-treatment with methotrexate. Panel D. Trophoblastic vascularization on color Doppler on day 5 post-treatment with methotrexate. **Fig. S2.** Patient 2. Panel A. Gestational sac of 15.4 mm, present at the level of uterine isthmus, localised at the level of CS scar. Yolk sac present. Embryo not seen. Panel B. Thick walls of gestational sac with increased peri-trophoblastic vascularization on color Doppler. Panel C. TVUS surveillance 5 days later. Persistent gestational sac at the isthmus, uterine cavity with blood content. Panel D. TVUS surveillance day 5. Persistent vascularization at the gestational sac-myometrium interface on color Doppler. **Fig. S3.** Patient 2. Panel A. Gestational sac persistent at re-admission, 10 days after the initial hospital discharge. Yolk sac present. Panel B. Present peri-trophoblastic vascularization on color Doppler. Embryo present. Panel C. Gestational sac retrieved by D&C. Panel D. Foley catheter was inserted at the level of uterine isthmus to tamponade the site of pregnancy implantation. **Fig. S4.** Patient 3. Panel A. Gestational sac localized at the level of CS scar. Panel B. Embryo present within gestational sac. Panel C. Embryo with cardiac activity present. Panel D. Day 8 after the therapy was initiated, the gestational sac and embryo were still present, showing peri-trophoblastic vascularization on color Doppler. Panel E. TVUS at 6 weeks showing an empty uterus and normal appearance of the CS scar at the isthmus. **Fig. S5.** Patient 4. Panel A. TVUS day 1 showing heterogeneous uterine content. A gestational sac was not seen at this time. Panel B. Gestational sac present. No embryo seen on day 3 of surveillance. Panel C. Day 10 of surveillance showing pregnancy in resolution. Panel D. TVUS 1 month after discharge showing resolution of scar pregnancy. **Fig. S6.** Patient 5. Panel A. TVUS day 1 showing a gestational sac with an embryo located at the level of previous CS scar. Panel B. Doppler color showing the peri-trophoblastic rich vascularization. Panel C. Day 7 of surveillance showing persistence of gestational sac. Panel D. Doppler color day 7 of surveillance showing persistence of scar pregnancy. Panel E and F. TVUS at 2 years from the CSP showing a normal anterior wall with normal appearance of the CS scar. **Fig. S7.** Patient 6. Panel A. TVUS on admission day showing a gestational sac with an embryo located at the level of previous CS scar. Panel B. TVUS post curettage showing a hyperechoic, heterogeneous mass (retained products of conception) located within the isthmus, at the level of previous CS scar. Doppler color showing persistence of vascularization within the mass. Panel C. TVUS post curettage showing an empty uterine cavity. Panel D. Two weeks follow up TVUS showing a persistent and growing mass at the isthmic level. Panel E. Doppler color showing persistence of intense vascularity around the remnant isthmic mass. **Fig. S8.** Patient 6. Panel A. Appearance of the uterus at the laparotomy. Note normal size of the uterus and bladder high on the isthmus, adherent post CS. Panel B. Appearance of the uterus, bulging isthmo-cervical region and both adnexa at laparotomy, after lysis of bladder adhesions. Panel C. Characteristics features of invasive placenta seen after further dissection.

## Data Availability

The datasets used and/or analysed during the current study available from the corresponding author on reasonable request.

## Abbreviations

CS: Caesarean section
CSP: Caesarean scar pregnancy
TVUS: Transvaginal ultrasound
hCG: Hormone chorionic gonadotroph
D&C: Dilation and curettage.
