# Supplementary material for: Combination of medical and surgical management in successful treatment of caesarean scar pregnancy: a case report series
Source: BMC Pregnancy Childbirth. 2020 Oct 13;20:617. doi: 10.1186/s12884-020-03237-8 (PMC7557042; doi:10.1186/s12884-020-03237-8)
Supplement: Supplementary file 1 — Additional file 1. Clinical Vignettes. This file includes detailed description of the six case reports. [file 12884_2020_3237_MOESM1_ESM.docx]

**Case presentation vignettes**

**Patient 1** was a 36-years old G3P2, who had two previous CS deliveries. She presented with abdominal pain, vaginal bleeding, and a history of 6-weeks amenorrhoea (Table 1). On examination, the patient was stable haemodynamically. Her abdomen was soft on palpation but mildly tender suprapubic. Vaginal examination revealed changes of pregnancy, with purple vaginal and exocervical mucosa, a small amount of dark blood in the vagina, cervix with os closed, and the uterus slightly enlarged, tender on bimanual palpation (Table 2). The TVUS findings, presented in detail in Table 3, showed a gestational sac localised within the CS scar area (see also Suppl Figure 1, Panel A). Colour Doppler showed a thick walled, richly vascularized, transonic image of 32 mm in diameter, bulging from the isthmus, leaving an imprint into the surrounding myometrium (Suppl Figure 1, Panel B). The initial hCG at admission was 23,100 IU/L (Table 1). Medical management was initiated, and the patient received a total of 5 injections of Methotrexate at a 1mg/kg per dose in alternative days. The falling trend in hCG and TVUS surveillance on day 5 post-treatment are shown in Table 4 and Suppl Figure 1, Panels C and D, respectively. The patient was discharge on request on day 9 in good condition, with vitals within normal range and no hypogastric pain or vaginal bleeding. She was followed up with weekly hCG until day 98, when the hCG levels were not detected (Table 3). TVUS surveillance at three months post admission revealed a normal uterus, with a normal appearance of the isthmic caesarean scar (Suppl Figure1 Panel E).

**Patient 2** was a 32-years old G6P4, who had four CS deliveries and one pregnancy termination. She presented to our unit for moderate hypogastric pain, vaginal bleeding moderate flow, no clots, and history of 8-weeks amenorrhoea (Table1). Vaginal examination revealed a closed multiparous cervix, with a small amount of dark blood coming through the external os. On bimanual examination the uterus was enlarged, 6-weeks in size, slightly tender (Table2). On TVUS, the gestational sac of 20.0 x11.0 mm in size had a yolk sac of 4mm and was localized at the isthmus, in the postoperative caesarean uterine scar, with deep protrusion into the anterior uterine wall. The myometrial wall measured 2.6 mm between gestational sac and bladder wall. (Suppl Figure 2, Panel A and B). On admission, the hCG level was 13,229 mUI/mL and Hb was 12.9g/dL (Table 3). The management options were discussed with the patient, and a decision for medical management was considered. Methotrexate at a dose of 1mg/kg body weight was initiated with serial monitoring of hCG levels at 48 h. Although the hCG levels decreased to 4,908 mUI/mL on day 9 of treatment, the hCG levels increased again to 16,626 mUI/mL on day 14^th^. The TVUS scan repeated in day 5 is shown in Suppl Figure 2, Panel C and D. The patient self-discharged but returned ten days later with vaginal bleeding and moderate hypogastric pain. An TVUS done at re-admission showed persistence of the gestational sac with no changes in local trophoblastic vascularization (Suppl Figure 3, Panel A and B). However, this time an embryo was present, with a CRL of 1.9 mm and no heart rate. Surgical management by aspiration curettage was done in the day of re-admission (Suppl Figure 3, Panel C). A Foley catheter was inserted at the isthmus and maintained for 2 days to ensure haemostasis (Suppl Figure 2, Panel D). There were no further complications and the patient was discharged on day 3 with follow up. At the time when this study was designed, the patient was still on surveillance, the hCG levels showing a lowering trend.

**Patient 3** was a 31-years old G2P1, who had one previous CS delivery, and presented with vaginal bleeding, pelvic pain, and history of 5-weeks amenorrhoea (Table1). On presentation, the patient was stable haemodynamically. Vaginal examination revealed a small amount of dark, old blood in the vagina, closed cervix, and slightly enlarged uterus, tender on palpation. On TVUS, the size of the uterus was 69.0 x 48.0 x 65.0 mm. A gestational sac of 20.3 mm protruding into the anterior uterine wall, at the level of the previous CS scar. A live embryo with CRL corresponding to a gestational age of 5-weeks 6-days was present within the sac (Table 2 and Suppl Figure 4, Panel A, B, C). At admission, the hCG level was 19,148 IU/L (Table 1). A medical management protocol was initiated with Mifepristone (dose 600mg orally), followed by administration of Methotrexate at a dose of 1mg/kg body weight IM. TVUS performed 8 days later showed persistent gestational sac with reduced peri-trophoblastic vascularization (Suppl Figure 4, Panel D and E). Serial hCG testing showed an initial increase followed by a decrease of the hormone levels (Table 4). Miscarriage ensued on day 10 with heavy uterine bleeding. A dilatation and vacuum-aspiration under ultrasound guidance was attempted to remove the pregnancy. A utero-vaginal pack was inserted for haemostasis and maintained for 12 hours under antibiotic protection. The following day after removal of the haemostasis pack, the patient was discharged from the hospital in good conditions, with no further bleeding or abdominal pain. Laboratory and US follow up surveillance showed resolution of pregnancy. Suppl Figure 4 Panel F shows an empty uterus with normal appearance of the uterine scar at 6 weeks post treatment (see also Table 3).

**Patient 4** was a 32-years old woman G4P3, with a history of three CS deliveries (Table 1) who presented in emergency with history of 3-days vaginal bleeding with fresh blood and clots on a background of 12-weeks of amenorrhoea. On admission, hCG level was 1,549 mUI/mL (Table 1). On TVUS, the uterus was retroverted, with heterogeneous content suggesting blood clots. With a presumptive diagnosis of pregnancy of unknow location conservative management with watchful surveillance was initiated. On day 3 from admission, hCG showed a moderate increase to 1,922 mUI/mL and a gestational sac was identified on TVUS, which was located at the level of the isthmus, implanted within the myometrium of the anterior wall, abutting posterior wall of the bladder. The TVUS findings met the criteria for a CS scar pregnancy (Table 2). A medical management protocol was initiated with Mifepristone 600 mg and Methotrexate 1 mg/kg as per local protocol. The patient was followed up as inpatient for 10 days and discharged in good conditions, with minimal genital bleeding and pain free (Table 4). TVUS showed a stationary ultrasound appearance (Suppl Figure 5 Panel C). TVUS surveillance showed resolution of scar pregnancy a month after hospital discharge (Suppl Figure 5 Panel D and Table 3).

**Patient 5** was a 23-years old woman, G3P2, with history of two previous CS deliveries (Table 1). She was diagnosed with intrauterine dichorionic diamniotic twin pregnancy of 7-weeks gestational age one week before presentation and underwent a termination of pregnancy at another institution. Two days after the procedure, she presented to maternity emergency triage with persistent, heavy vaginal bleeding and lower abdominal cramping. On examination, the bleeding was moderate, the cervix was normal, and the uterus was slightly increased in size and tender. Cervical excitation was present (Table 2). On admission, the level of beta hCG was 4,000 mUI/mL and Hb was 12.1 g/dL (Table 1). TVUS showed an anteverted uterus with inhomogeneous content and a 17 mm gestational sac located at the level of uterine isthmus, implanted deeply within the anterior myometrium. A yolk sac and embryo were present, although no cardiac activity was identified (Table 2 and Suppl Figure 6, Panel A). Color Doppler showed intense vascularity around the gestational sac (Suppl Figure 6, Panel B). The patient chose surgical management as first intention and an uncomplicated D&C was done on the day of admission. Follow up showed a drop in hCG levels to 1,440 mUI/mL in day 1 post curettage. Due to persistence of vascularized trophoblastic tissue seen 7 days post-surgery on TVUS (Suppl Figure 6, Panel C and D), the Methotrexate protocol was initiated at a dose of 1mg/kg a dose, administered IM. Two days later, the patient had heavy vaginal bleeding that precluded administration of the 2^nd^ dose of Methotrexate and required emergency surgical intervention with insertion of a Foley catheter at the isthmus for haemostasis. A drop in Hb levels to 8.9 g/dL required administration of an infusion with iron IV. Upon catheter removal, the patient was discharged home in good conditions. Two years from the ectopic scar pregnancy, the sonographic evaluation of the uterus revealed a normal anterior wall, with normal appearance of the caesarean scar.

**Patient 6** was 28 years old G2P1 with one previous delivery by CS (Table 1). The patient’s personal medical history was relevant for AIDS stage 3 and pulmonary tuberculosis, both contracted as teenager and adequately treated. On presentation, the patient reported a history of amenorrhea of 13weeks and 6 days, persistent lower abdomen pain, and vaginal spotting. Vaginal examination showed no evidence of vaginal bleeding. The cervix was long and closed, particularly tender, cervical excitation was present. The uterus was increased in size of approximately 6-7 weeks, thus discordant to the duration of the amenorrhea. The uterus was tender, and the adnexa were non-palpable and non-tender bilaterally (Table 2). On admission, laboratory tests showed a Hb of 12.4 g/L and hCG 397 mUI/mL (Table 1). TVUS found an irregular gestational sac of 23.3 mm, located low within the uterine cavity, at the level of isthmus. An embryo with CRL of 14.8 mm, corresponding to a pregnancy of 7 weeks and 6 days, with absent cardiac activity was present within the sac (Table 2 and Suppl Figure 7, Panel A). Color Doppler showed intense vascularization retro vesical, within the anterior uterine wall. Within hours from admission, before the medical management was initiated, a sudden onset of heavy bleeding dropped the Hb to 7.5 g/L and required a salvage D&C, that retrieved copious amount of fibrotic tissue, very adherent. A Foley catheter was inserted for 24 h for haemostasis. The patient received one unit of red blood cells and one unit of fresh frozen plasma. The TVUS post curettage showed a uterus of 82x63x66 mm with a hyperechoic, heterogeneous mass of 45x37 mm located within the isthmus, at the level of previous CS scar (Suppl Figure7, Panel B). The uterine cavity was empty (Suppl Figure7, Panel C). The patient was discharged with serial hCG surveillance (Table 4). Two weeks later aTVUS showed an empty uterus and increase in size and intense vascularization of the isthmic mass (Suppl Figure 7, Panel D and E), with high index of suspicion on invasive placentation. The possibility of accreta was supported by an MRI examination, which showed the uterine mass protruding through the dehiscent uterine wall towards the posterior wall of the bladder, abutting but not invading the bladder wall. A diagnosis of trophoblastic neoplasia was also entertained. In view of these findings the patient underwent a total hysterectomy. At the surgery was found that the cervico-isthmic mass was protruding through the uterine wall, whose integrity was compromised but did not progress into the posterior balder wall (Suppl Figure 8, Panel A, B, C). The histology confirmed presence of necrotic chorionic villi and inflammation but no findings of trophoblastic invasion beyond the uterine serosa.
