# Supplementary figures and images for: Combination of medical and surgical management in successful treatment of caesarean scar pregnancy: a case report series
Source: BMC Pregnancy Childbirth. 2020 Oct 13;20:617. doi: 10.1186/s12884-020-03237-8 (PMC7557042; doi:10.1186/s12884-020-03237-8)

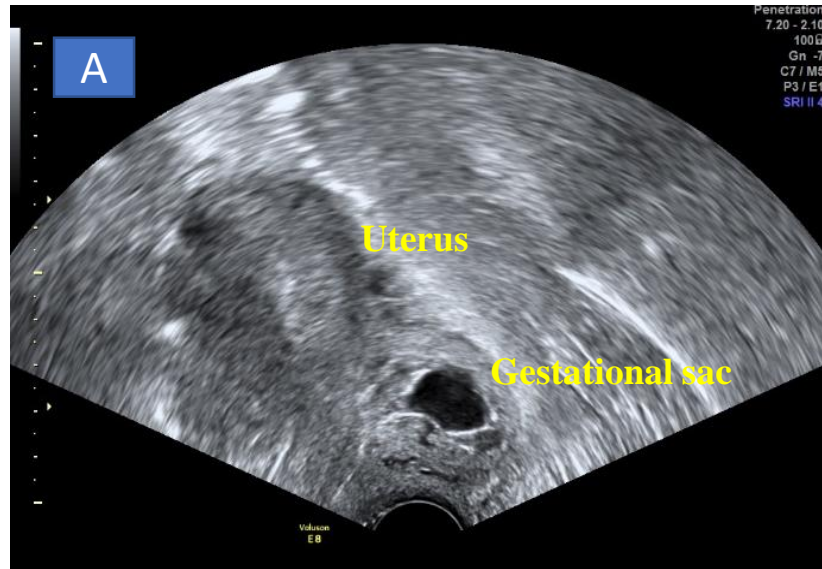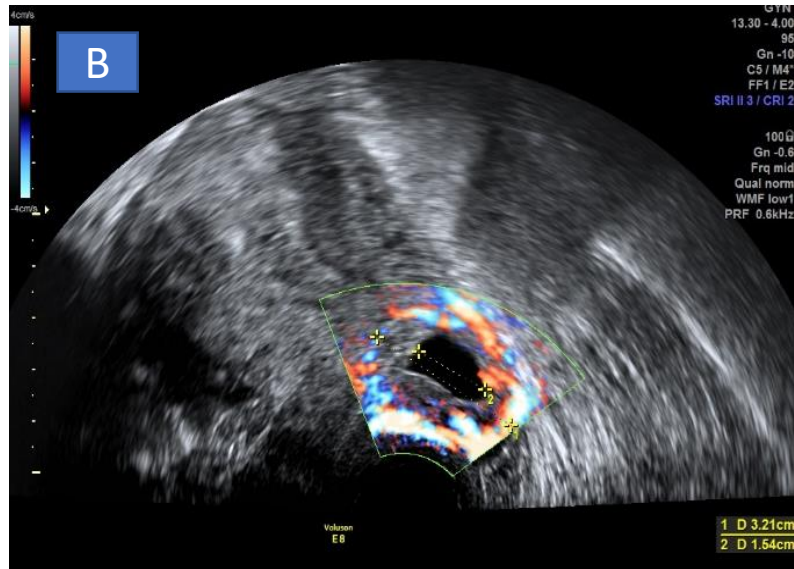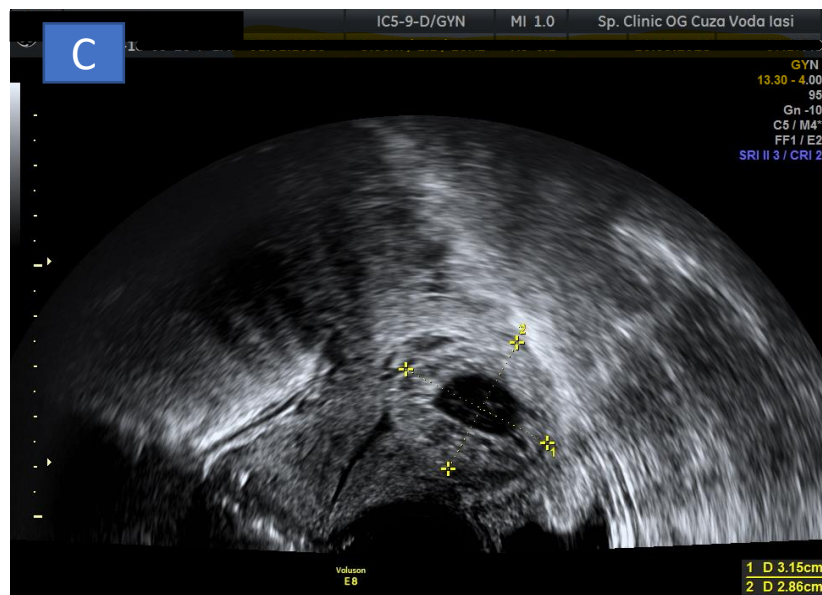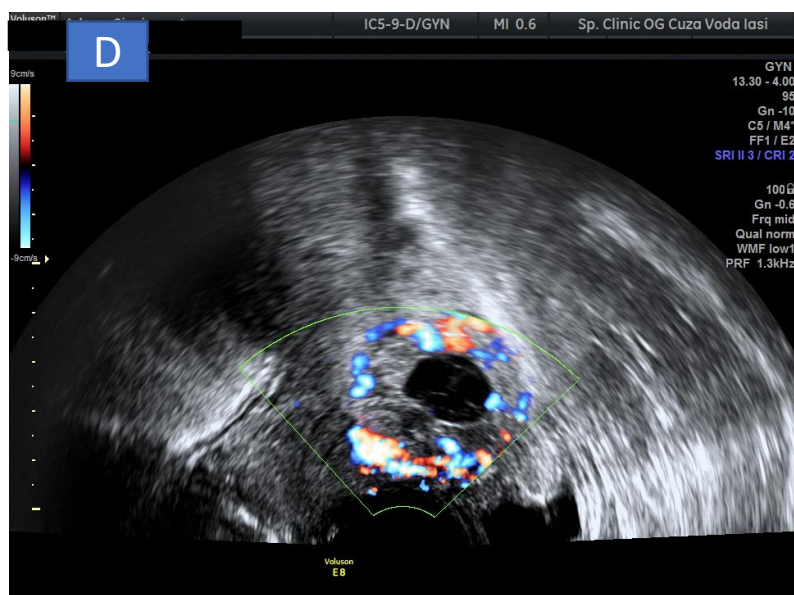

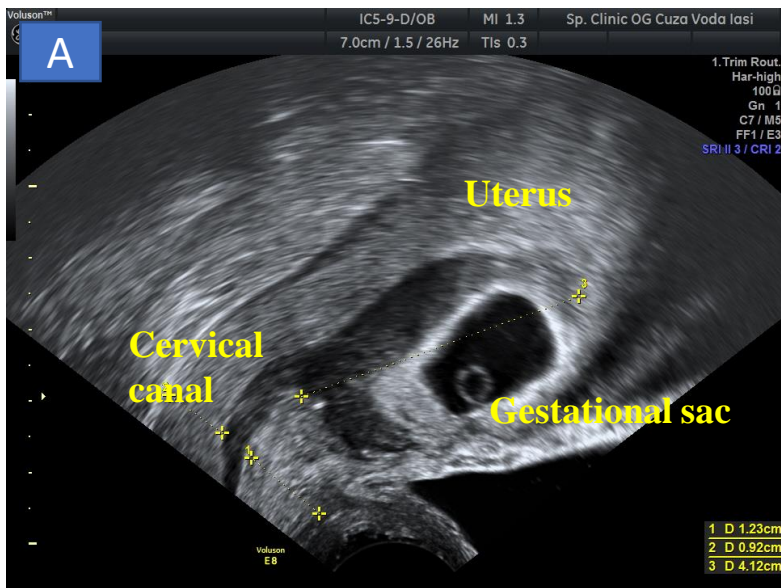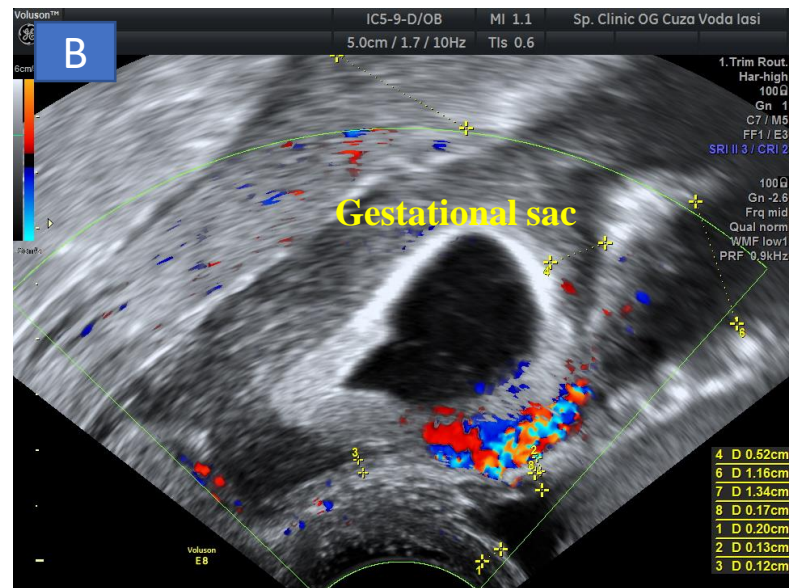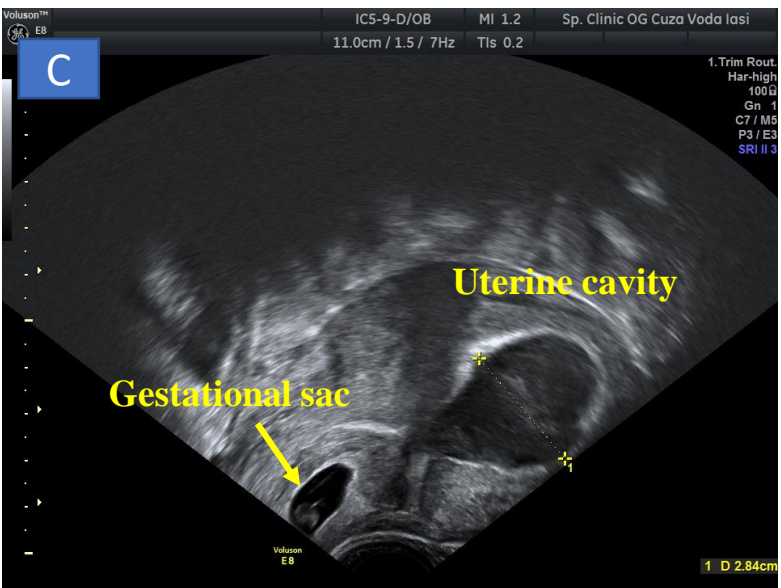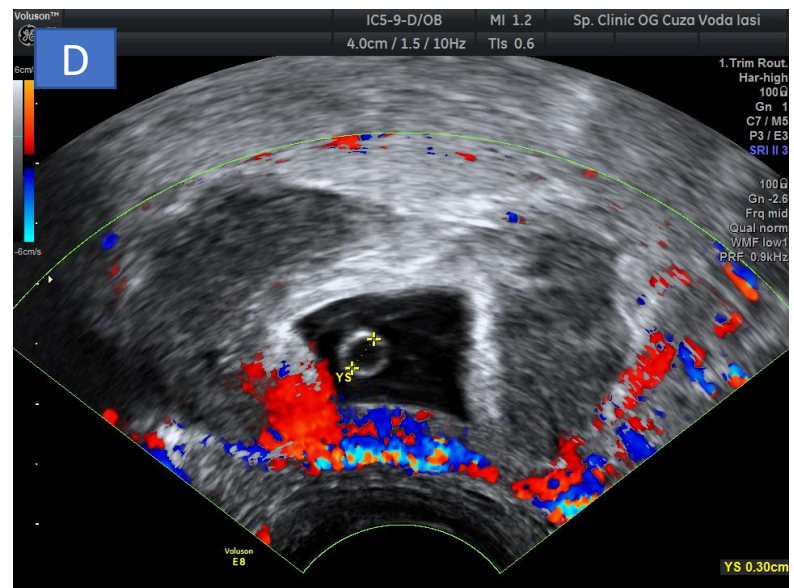

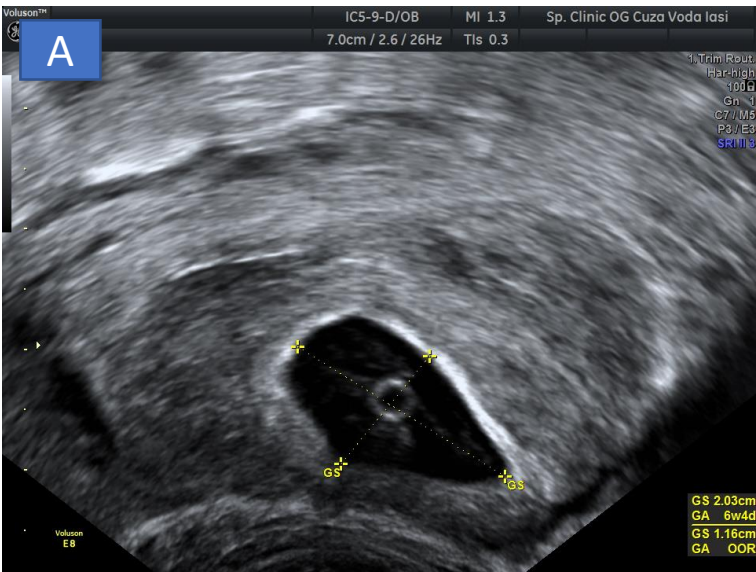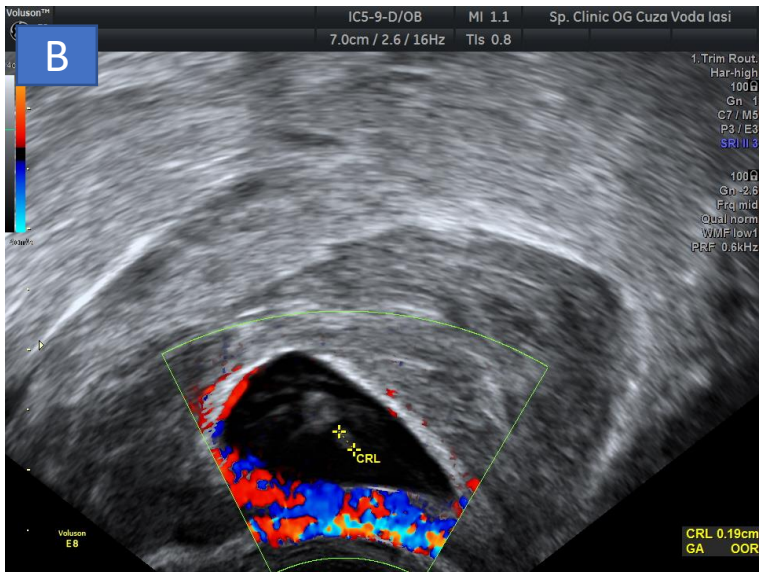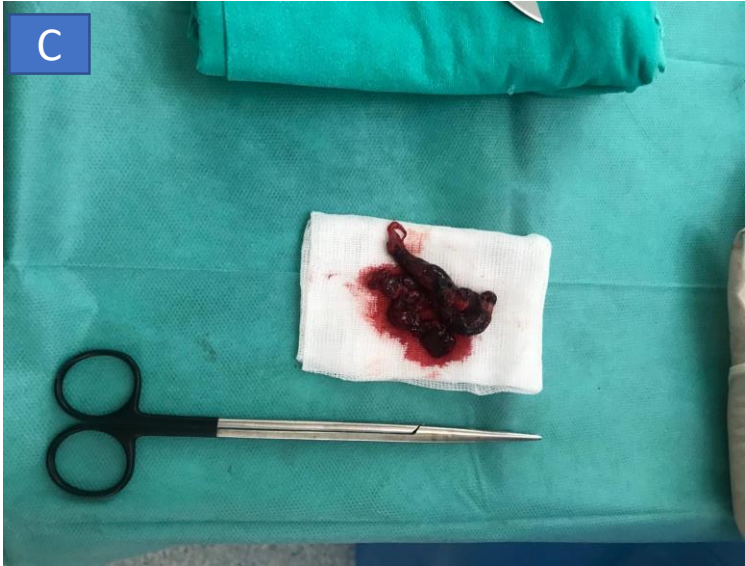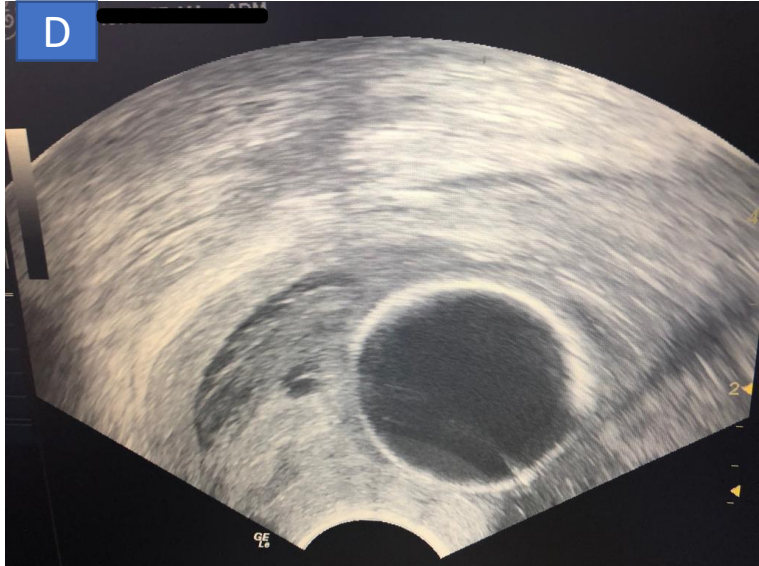

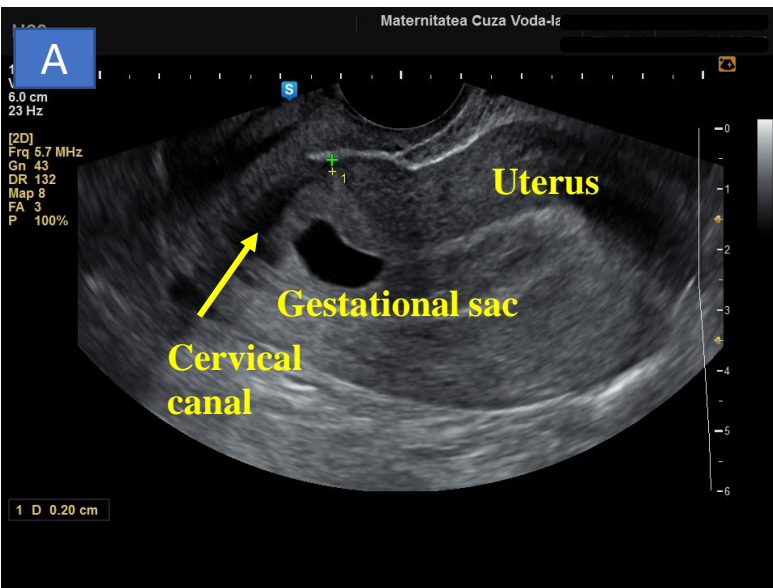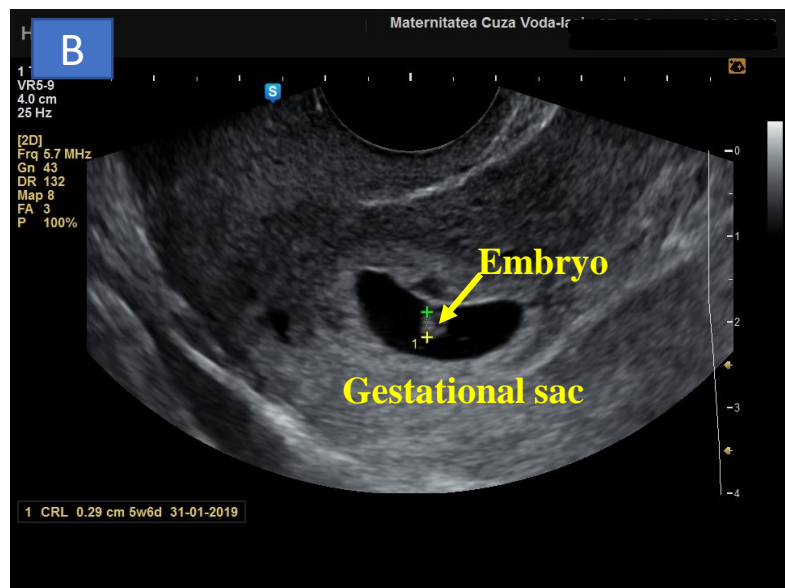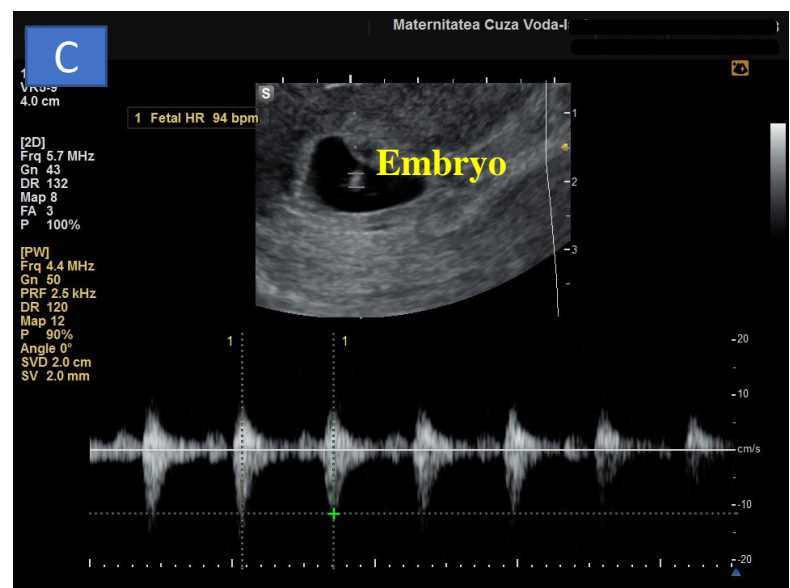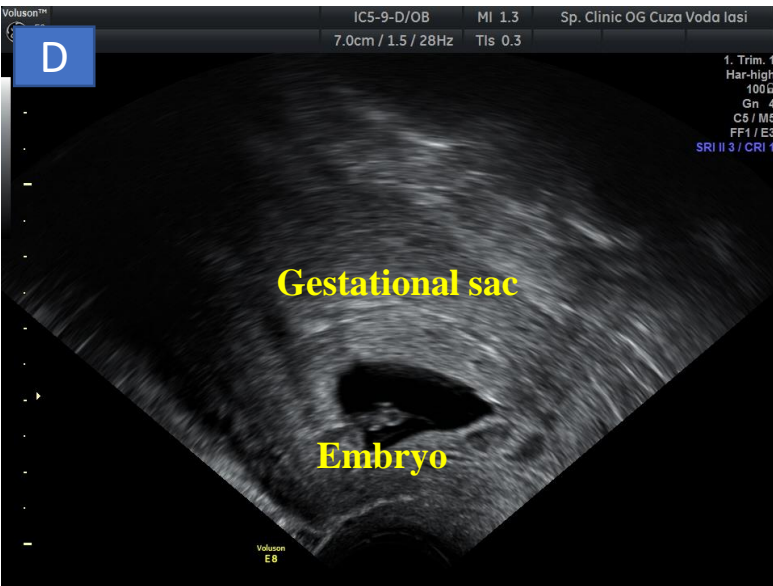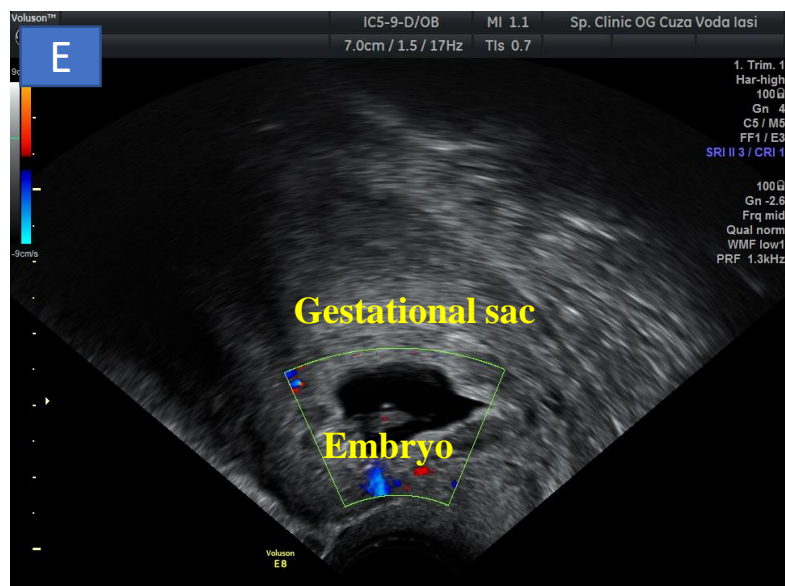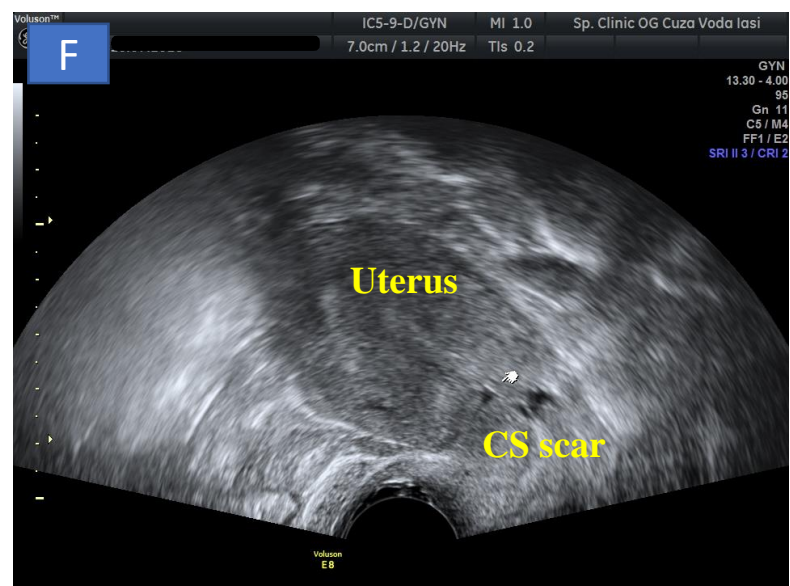

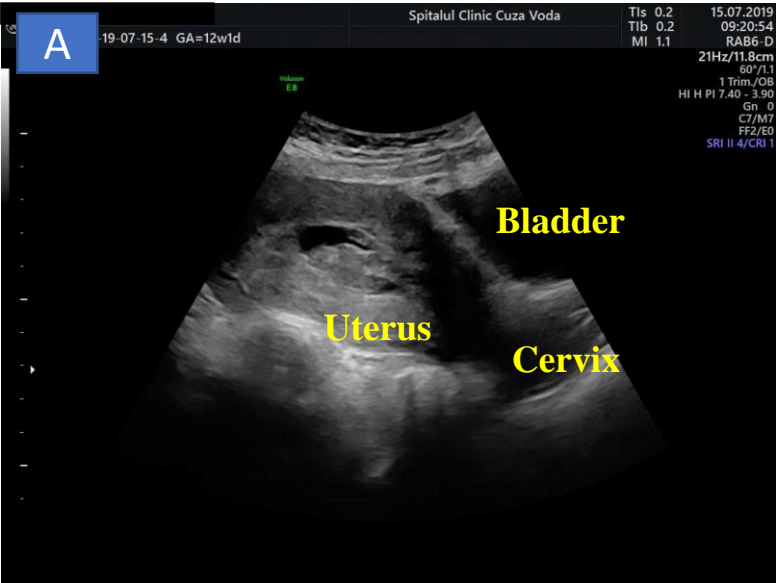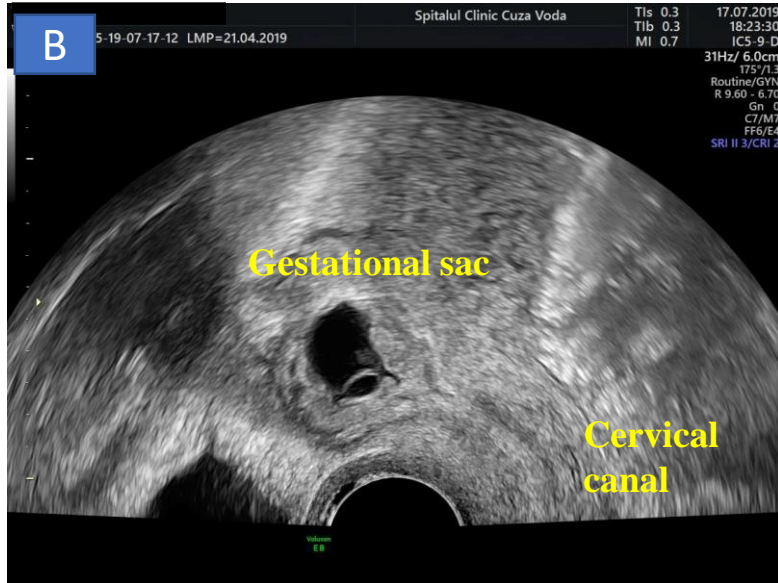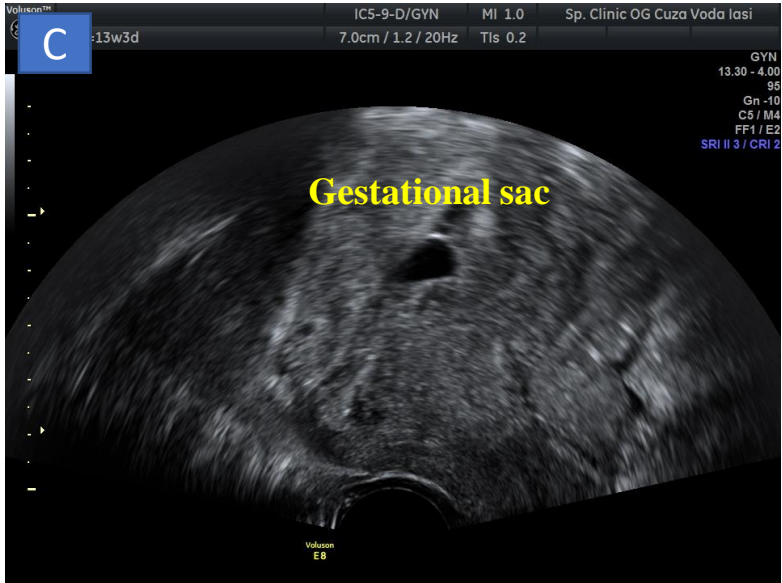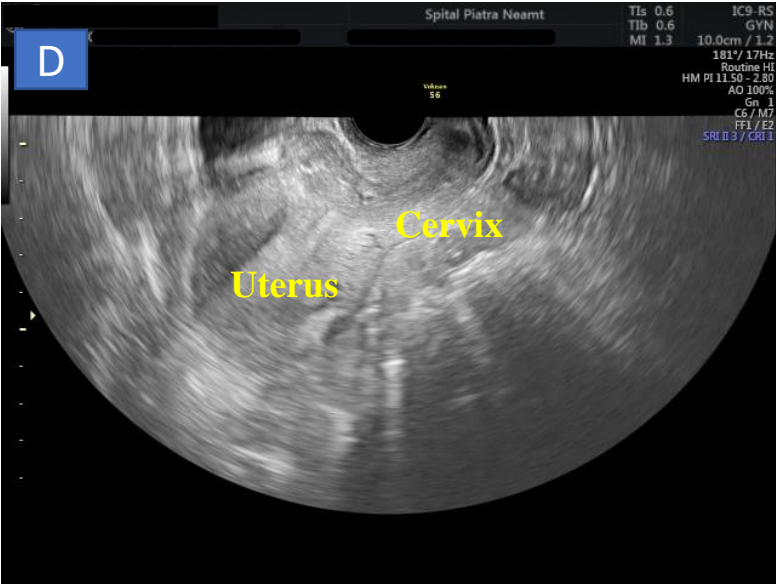

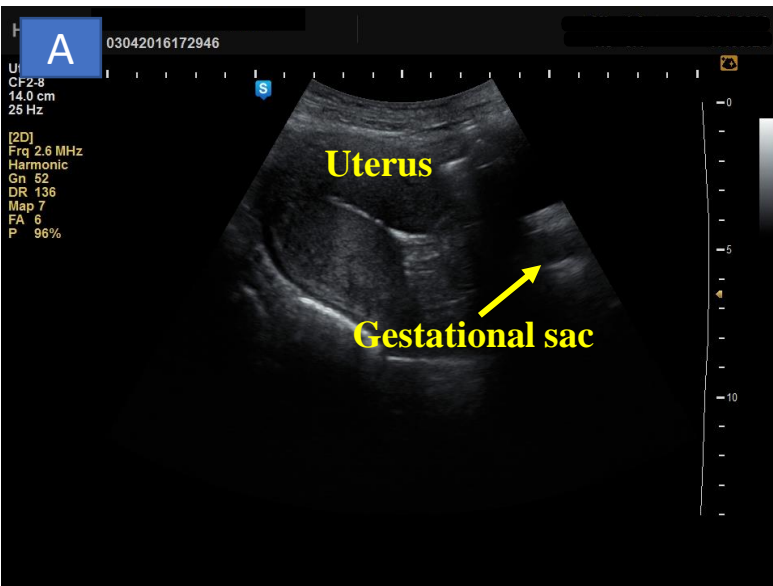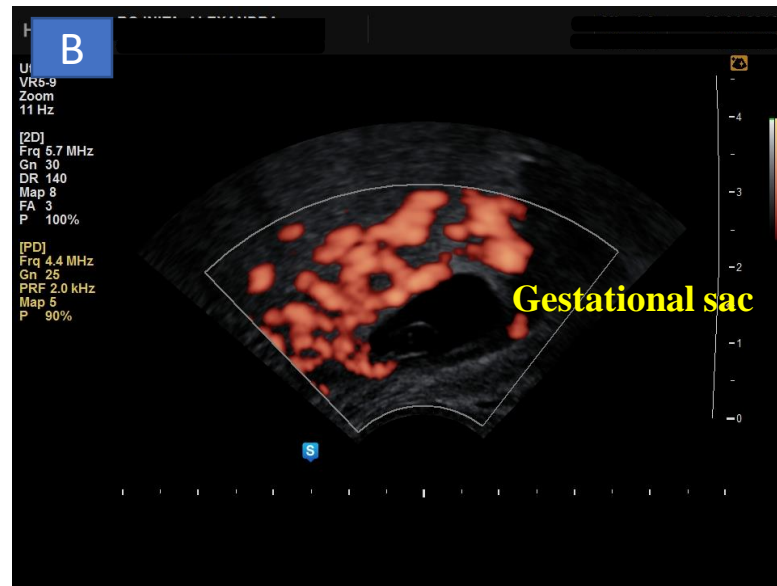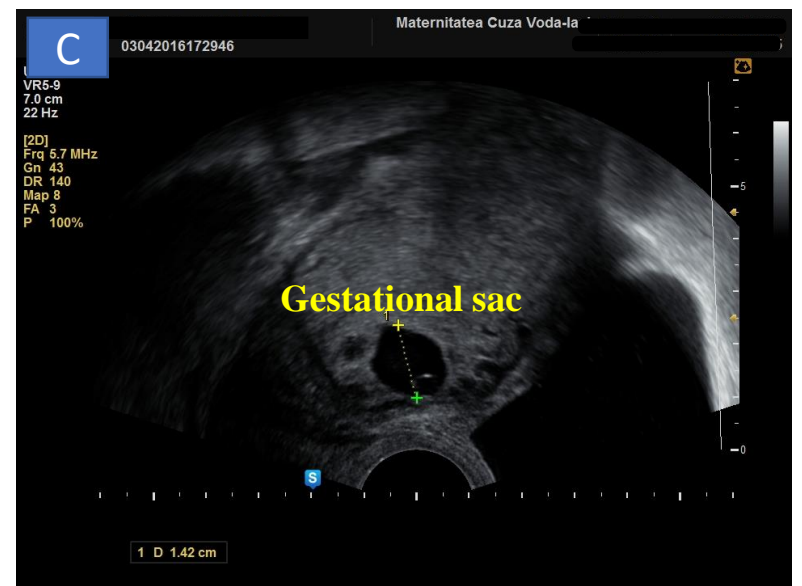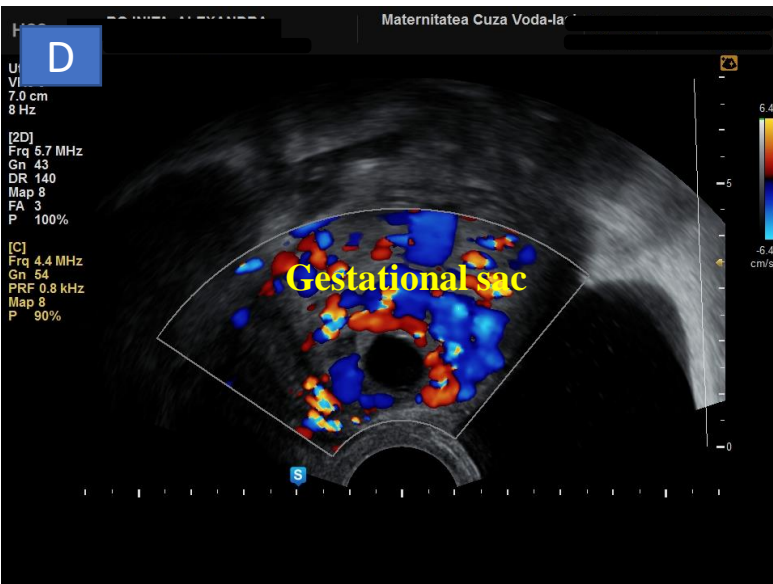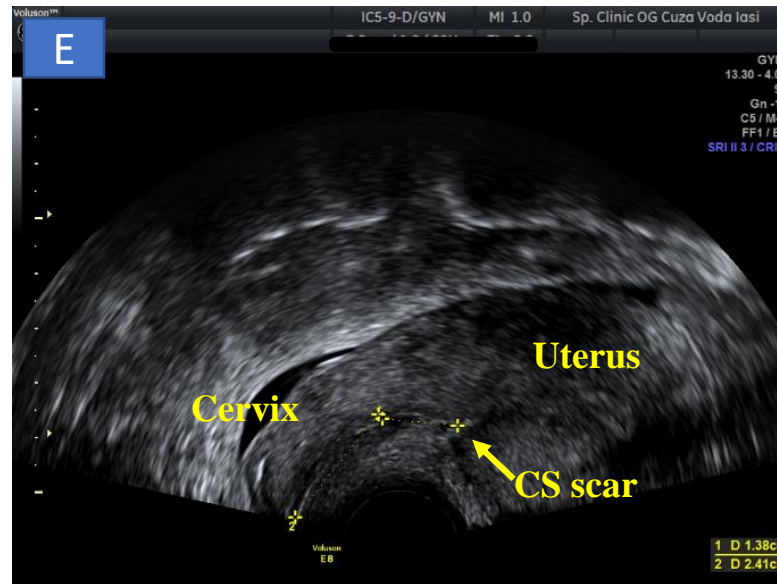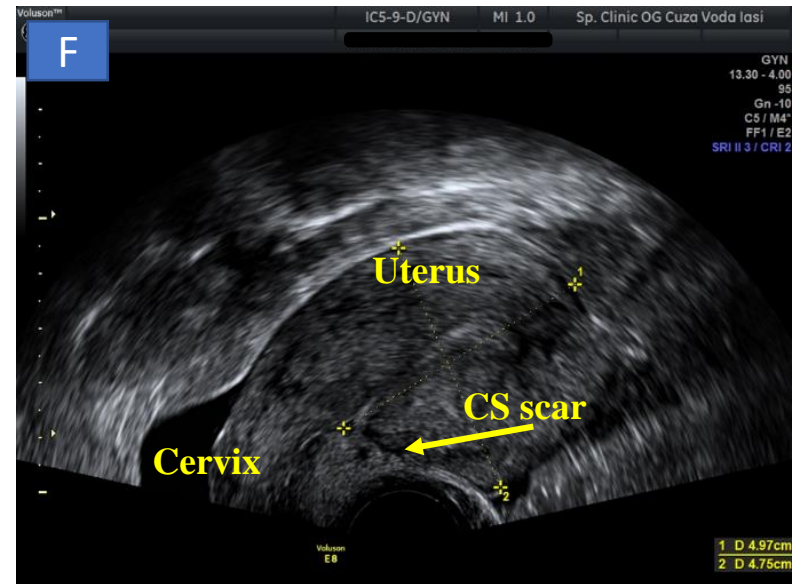

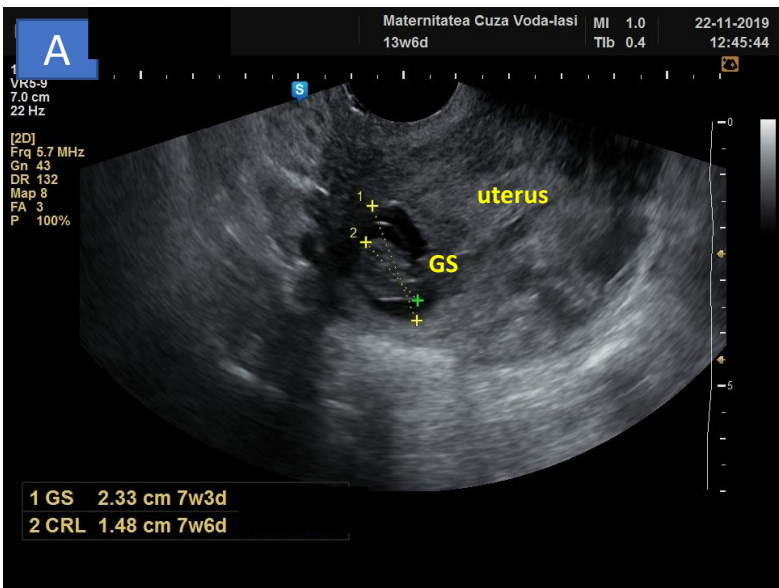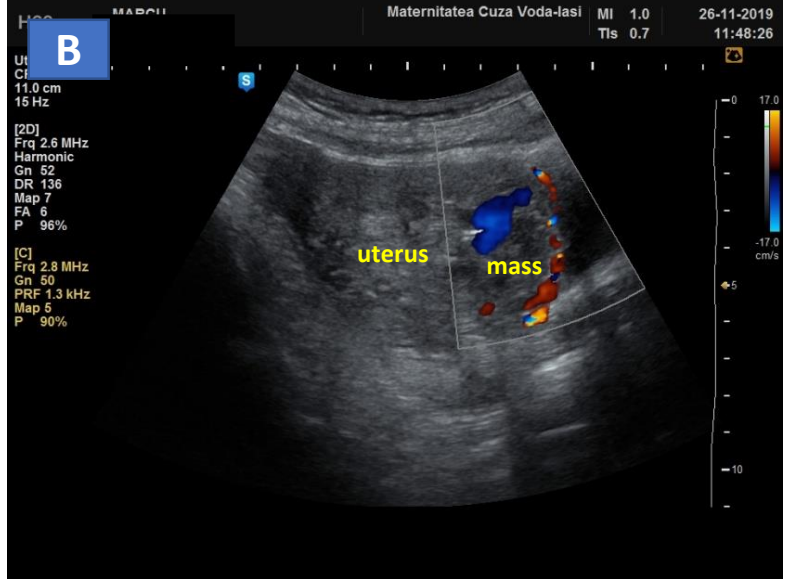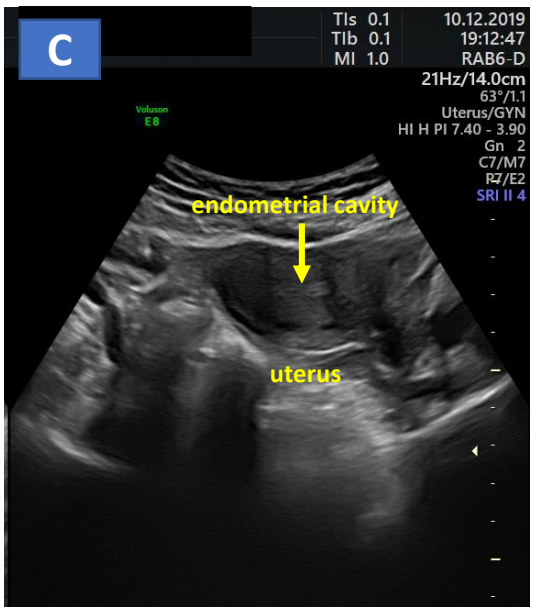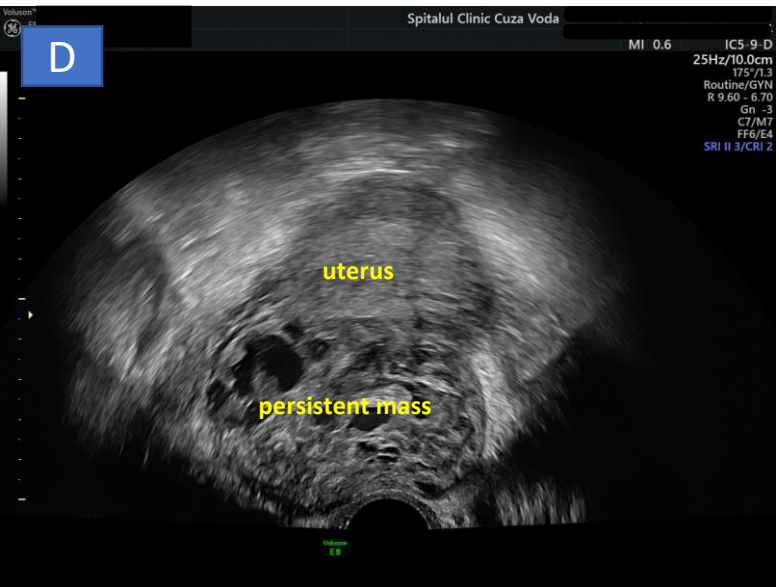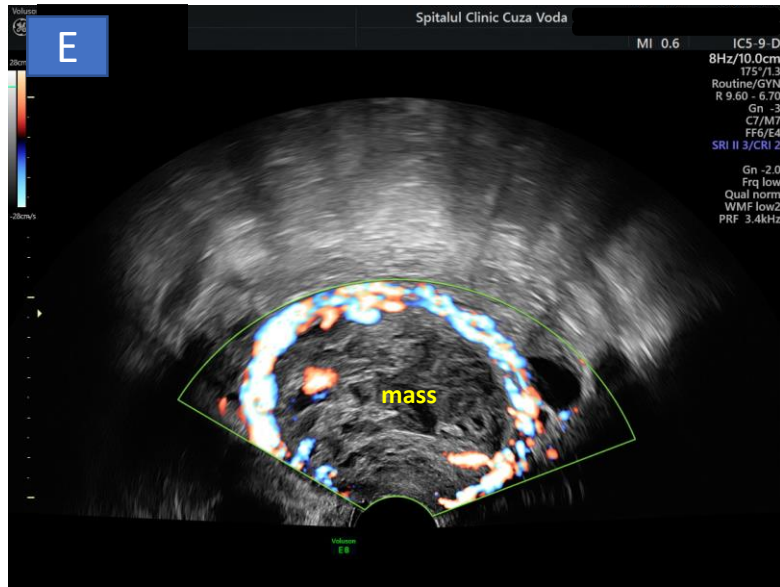

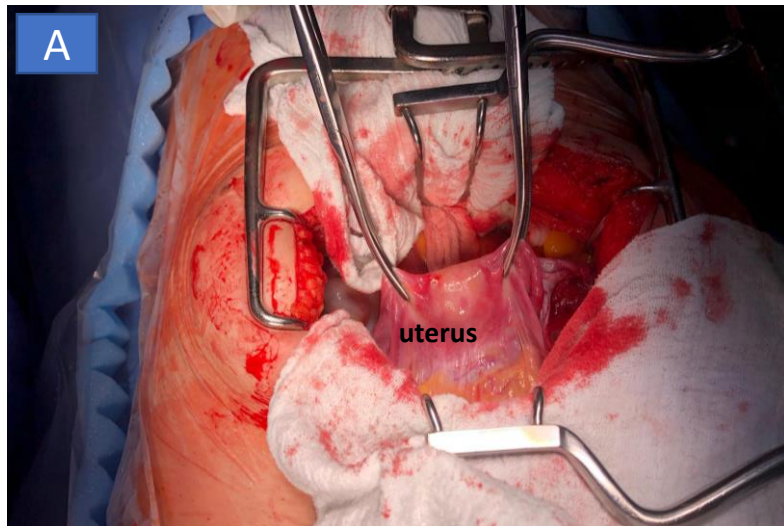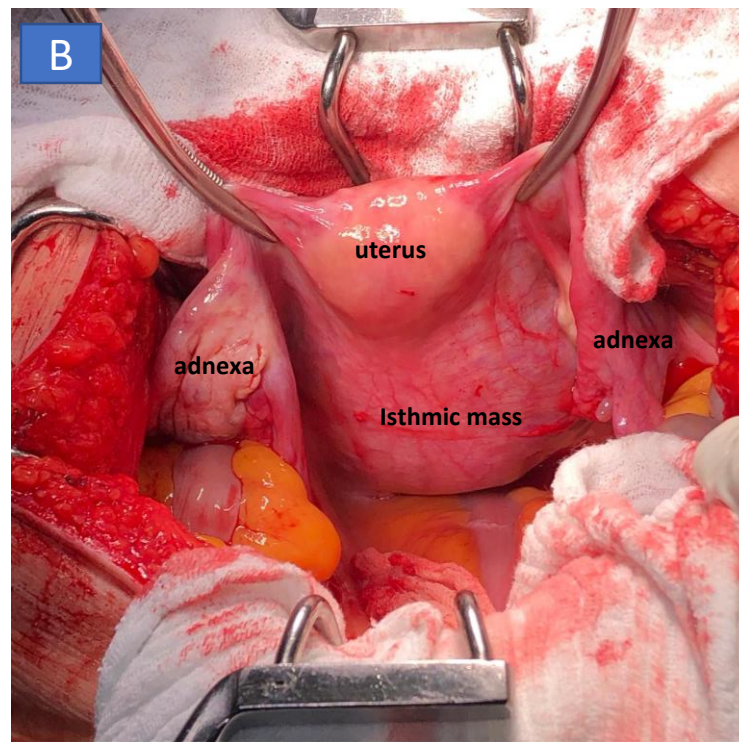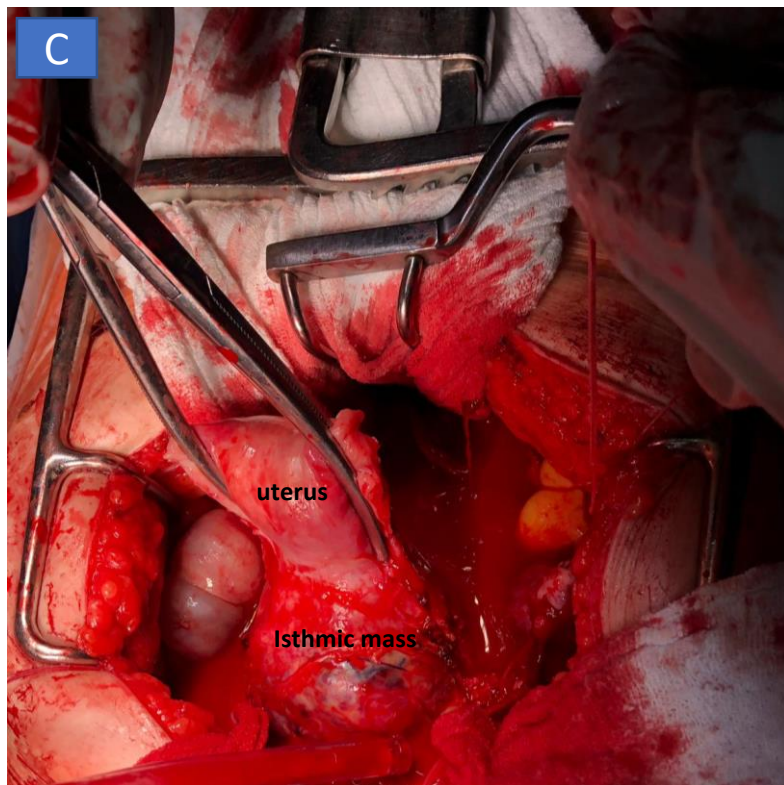

Supplement: Supplementary file 2 — Additional file 2 Fig. S1. Patient 1. Panel A. Gestational sac present at the level of uterine isthmus, localised within CS scar. Embryo not seen. Panel B. Increased peri-throphoblastic vascularization on color Doppler. Panel C. Gestational sac still present day 5 post-treatment with methotrexate. Panel D. Trophoblastic vascularization on color Doppler on day 5 post-treatment with methotrexate. Fig. S2. Patient 2. Panel A. Gestational sac of 15.4 mm, present at the level of uterine isthmus, localised at the level of CS scar. Yolk sac present. Embryo not seen. Panel B. Thick walls of gestational sac with increased peri-trophoblastic vascularization on color Doppler. Panel C. TVUS surveillance 5 days later. Persistent gestational sac at the isthmus, uterine cavity with blood content. Panel D. TVUS surveillance day 5. Persistent vascularization at the gestational sac-myometrium interface on color Doppler. Fig. S3. Patient 2. Panel A. Gestational sac persistent at re-admission, 10 days after the initial hospital discharge. Yolk sac present. Panel B. Present peri-trophoblastic vascularization on color Doppler. Embryo present. Panel C. Gestational sac retrieved by D&C. Panel D. Foley catheter was inserted at the level of uterine isthmus to tamponade the site of pregnancy implantation. Fig. S4. Patient 3. Panel A. Gestational sac localized at the level of CS scar. Panel B. Embryo present within gestational sac. Panel C. Embryo with cardiac activity present. Panel D. Day 8 after the therapy was initiated, the gestational sac and embryo were still present, showing peri-trophoblastic vascularization on color Doppler. Panel E. TVUS at 6 weeks showing an empty uterus and normal appearance of the CS scar at the isthmus. Fig. S5. Patient 4. Panel A. TVUS day 1 showing heterogeneous uterine content. A gestational sac was not seen at this time. Panel B. Gestational sac present. No embryo seen on day 3 of surveillance. Panel C. Day 10 of surveillance showing pregnancy in resolution. [file 12884_2020_3237_MOESM2_ESM.pdf]
